# Burden and Characteristics of the Comorbidity Tuberculosis—Diabetes in Europe: TBnet Prevalence Survey and Case-Control Study

**DOI:** 10.1093/ofid/ofy337

**Published:** 2018-12-19

**Authors:** Monica Sane Schepisi, Assunta Navarra, M Nieves Altet Gomez, Andrii Dudnyk, Anne Margarita Dyrhol-Riise, Jaime Esteban, Pier Francesco Giorgetti, Gina Gualano, Lorenzo Guglielmetti, Jan Heyckendorf, Anna Kaluzhenina, Berit Lange, Christoph Lange, Katerina Manika, Jalal Miah, Zorica Nanovic, Emanuele Pontali, Monica Rios Prego, Ivan Solovic, Simon Tiberi, Fabrizio Palmieri, Enrico Girardi

**Affiliations:** 1 Clinical Epidemiology Unit, National Institute for Infectious Diseases L. Spallanzani – IRCCS, Rome, Italy; 2 Unidad de Tratamiento Directamente Observado de la Tuberculosis “Servicios Clínicos,” Barcelona, Spain; 3 Tuberculosis, Clinical Immunology & Allergy Department, National Pirogov Memorial Medical University, Vinnytsia, Ukraine; 4 Department of Infectious Diseases, Oslo University Hospital, Institute of Clinical Medicine, University of Oslo, Department of Clinical Science, University of Bergen, Norway; 5 Departamento de Microbiología Clínica, Fundación Jiménez Díaz, Madrid, Spain; 6 Clinica di Malattie Infettive e Tropicali, A. O. Spedali Civili di Brescia e Università di Brescia, Brescia, Italy; 7 Clinical Department, National Institute for Infectious Diseases L. Spallanzani – IRCCS, Rome, Italy; 8 Sanatorium, Centre Hospitalier de Bligny Briis-sous-Forges, Paris, France; 9 APHP, Centre National de Référence des Mycobactéries et de la Résistance des Mycobactéries aux Antituberculeux (CNR-MyRMA), Bactériologie-Hygiène, Hôpitaux Universitaires Pitié Salpêtrière-Charles Foix, Paris, France; 10 Sorbonne Université, Unité 1135, Team E13 (Bactériologie), CR7 INSERM, Centre d’Immunologie et des Maladies Infectieuses, Paris, France; 11 Research Center Borstel. German Center for Infection Research (DZIF), Borstel, Germany; 12 Department of Phthisiopulmonology, Volgograd State Medical University, Volgograd, Russian Federation; 13 Infectious Disease Division, Department of Medicine II, Medical Center – University of Freiburg, Faculty of Medicine, University of Freiburg, Germany; 14 Center for Chronic Immunodeficiency, Faculty of Medicine, Medical Center, University of Freiburg, Germany; 15 Respiratory Infections Unit, Pulmonary Department, Aristotle University of Thessaloniki, “G. Papanikolaou” Hospital, Thessaloniki, Greece; 16 Institute of Lung Diseases and Tuberculosis – Skopje, Institute of Lung Diseases and Tuberculosis – Skopje, Skopje, FYROM (Macedonia); 17 Divisione di Malattie Infettive, Ospedale Galliera – Genova, Genova, Italy; 18 Enfermedades Infecciosas, Medicina Interna, Complexo Hospitalario Universitario de Pontevedra, Pontevedra, Spain; 19 Catholic University Ruzomberok, Slovakia; 20 Division of Infection, Barts Health NHS Trust, London, United Kingdom

**Keywords:** diabetes mellitus, tuberculosis, foreign-born, Europe

## Abstract

**Background:**

The growing burden of diabetes mellitus (DM) is posing a threat to global tuberculosis (TB) control. DM triples the risk of developing TB, modifies the presenting features of pulmonary TB, and worsens TB treatment outcomes. We aimed to analyze the prevalence of DM among TB patients and to describe the characteristics and clinical presentation of TB-DM patients in Europe.

**Methods:**

We performed a cross-sectional survey on the prevalence of DM among consecutively diagnosed adult TB patients in 11 European TB referral centers located in France, Germany, Greece, Italy, Russia, Slovakia, Spain, and the United Kingdom over the period 2007–2015. We also selected DM-TB cases and TB only controls with a 1:3 ratio to perform a case-control analysis, including patients selected from the countries mentioned above plus Norway and Ukraine.

**Results:**

Among 3143 TB enrolled patients, DM prevalence overall was 10.7% and ranged from 4.4% in Greece to 28.5% in the United Kingdom. Patients’ median ages ranged from 36 to 49 years, and all centers had >60% males; the proportion of foreign-born patients varied widely across sites. In the case-control study, DM was independently associated with older age and, among older patients, with being foreign-born. Among patients with pulmonary involvement, cavities on chest imaging were more frequently observed among those with DM.

**Conclusions:**

Diabetes mellitus represents a challenge for TB control in Europe, especially in foreign-born and in elderly patients. Specific screening strategies should be evaluated.

In 2016, an estimated 290 000 new tuberculosis (TB) cases and relapses occurred in World Health Organization (WHO) European Region countries, equivalent to 31.6 cases per 100 000 population and representing about 3.0% of the total global burden of TB. During the last decade, the European Region achieved the fastest decline in the world compared with other WHO regions. Nevertheless, there is a need for an even quicker decline in TB incidence to meet the targets of End TB Strategy by 2035 [1]. In this context, it is essential to address population groups that present conditions that increase the risk of developing active TB disease [2].

Diabetes mellitus (DM) has recently re-emerged as a significant risk factor for TB. Five systematic reviews that aimed to quantify the increased risk of developing TB among people with type 2 diabetes published between 2008 and 2018 found that DM increases the likelihood of developing TB by 2- to 3-fold [3–7]. According to the WHO, about 15% of TB cases globally can be attributed to DM [8], and DM is now the second most important risk factor for TB in the central European region and other established market economies [9].

The number of people with DM in Europe in 2017 is estimated to be 58 (95% confidence interval [CI], 46.5–79.5) million (age-adjusted comparative prevalence, 6.8%; 95% CI, 5.4%–9.9%), including 22 million undiagnosed cases. Although the European Region has the second-lowest age-adjusted comparative DM prevalence rate, after the African Region, there are still many European countries with relatively high DM prevalence rates [10].

A systematic review published in 2017 has reported an overall global median DM prevalence among TB patients of 16% [11], ranging from 1.9% in Cotonou-Benin to 45% in the Ebeye-Marshall Islands [12, 13], a higher burden compared with the findings of the previous systematic review conducted in 2010 [14]. This recently published systematic review [11] retrieved only 7 studies conducted in low–TB incidence countries, of which only 2 were European [15, 16].

Other published studies on DM prevalence among TB patients conducted in Europe during the last 2 decades reported prevalence rates ranging from 3.2% in Palma de Mallorca (Spain) [17] to 14.6% in Finland [18]. A broader survey and a comprehensive analysis on the DM-TB comorbidity in Europe are not available in the literature. Thus, we have performed a study to assess DM prevalence among patients managed for TB in clinical centers across Europe and analyzed the sociodemographic and clinical characteristics of these patients.

## METHODS

### Study Participants and Study Design

We designed a retrospective cross-sectional survey and a case-control study aimed to assess the prevalence of DM among TB patients during the period 2007 to 2015 in Europe and to identify sociodemographic and clinical-radiological factors associated with DM. Members of the Tuberculosis Network European Trials group, a network of clinical centers active in the field of TB research in Europe, were invited to join the study (TBnet; http://www.tbnet.eu) [19].

### Retrospective Survey

We included all patients with TB from the participating TBnet centers for a period of 1 to 5 years during 2007–2015 depending on the local availability of data. Patients with DM were identified among patients with TB from clinical records and laboratory data already available at the participating TBnet centers. Patients included in this analysis had either a known diagnosis of DM or sufficient clinical or laboratory information to diagnose or exclude DM. DM cases were defined according to the following criteria: (1) previous diagnosis of DM type 1 or type 2 (patients with a physician-based diagnosis of DM before TB diagnosis and/or currently receiving treatment for DM) or (2) patients diagnosed with DM type 1 or type 2 at the time of TB diagnosis: (a) 2 fasting glucose tests >126 mg/dL or (b) 1 oral glucose tolerance test >200 mg/dL or (c) in a patient with classic symptoms of hyperglycemia or hyperglycemic crisis, a random plasma glucose level >200 mg/dL during TB treatment and (d) 1 HbA1c result >6.5 or fasting glucose level >126 mg/dL during TB treatment.

### Case-Control Study

For each case of DM-TB, the following 3 consecutive TB patients without DM, diagnosed in the same center, were selected as controls. DM-TB cases for which 3 controls could not be identified in the same clinical center were excluded from the analysis.

The following data were collected for patients included in this analysis: (1) sociodemographic and epidemiological information, including country of origin, age, gender, previous TB diagnosis, excessive alcohol intake, drug abuse, HIV status, comorbidities—including long-term steroids, cytotoxic treatment, immunosuppressive medications, chronic renal failure/liver disease/lung disease, hematological or other malignancies, silicosis, gastrectomy, anti–tumor necrosis factor treatment; (2) clinical data: respiratory and general symptoms, TB localization (pulmonary/extrapulmonary), radiological or computed tomography findings (location of lung lesions, presence of cavitary lesions), *Mycobacterium tuberculosis* microbiological examinations (smear microscopy and culture results), previous TB, DM diagnosis, plasma glucose, and glycated hemoglobin results. Regarding migration status, patients were classified as autochthonous (those born in the same country where TB was diagnosed) or foreign-born. Countries of birth were grouped according to prevalence of DM in the general population, as reported in the 2017 Diabetes Atlas [10].

Data were anonymized and exported in a common format from local databases, and a central database was created at the coordinating center.

### Statistical Analysis

Variables that met the *P* < .2 significance level at univariable analysis were retained for the multivariable model, which also incorporated standard sociodemographic variables. Two distinct multivariable models were fitted: we used conditional logistic regression to generate crude and adjusted odds ratios and 95% confidence intervals (CIs) of the association between (1) DM and sociodemographic/clinical characteristics among all TB patients and (2) DM and clinical/radiological presentation of TB among cases with pulmonary involvement. To take into account the possible correlation that may arise from observations belonging to the same center, in the conditional logistic regression model, robust standard errors were obtained using the option cluster “vce (cluster clustvar)” with the cluster identifying the TB diagnosis centers. We tested the hypothesis of interaction between variables in the model using the Bayesian Information Criterion (BIC). All statistical analyses were conducted using StataCorp 2013 (Stata Statistical Software, Release 13, StataCorp LP, College Station, TX).

### Ethics Board Approval

Authorization for the use of personal data for research purposes was first received from the Ethics Committee of the Coordinating Centre (INMI Lazzaro Spallanzani) according to the regulations of the Italian Data Protection Authority (“General Authorisation to Process Personal Data for Scientific Research Purposes,” March 1, 2012, as published in Italy’s Official Journal No. 72, dated March 26, 2012). Each participating center subsequently collected ethical approval according to national/local regulations on personal data protection.

## RESULTS

Thirteen clinical centers located in 10 different European countries—France (Briis-sous-Forges), Germany (Borstel), Greece (Thessaloniki), Italy (Rome and Genova), Norway (Oslo), Russia (Volgograd), Slovakia (Vysne Hagy), Spain (Barcelona, Madrid and Pontevedra), the United Kingdom (London), and Ukraine (Vinnytsia)—participated in this study. The clinical centers located in Ukraine and Norway provided data only for the case-control study, not for the prevalence study. One center reported using random plasma glucose level testing and HbA1c determination for screening TB patients for DM, whereas the others reported using random or fasting plasma glucose level. For DM diagnostic confirmation, all centers reported using HbA1c determination.

### Study Participant Characteristics

Overall, 3143 TB patients were included in the prevalence analysis (Table 1). The percentage of male patients was >60% in all centers, and patients’ median ages ranged from 36 to 49 years. Wide differences were observed in the proportion of foreign-born participants among centers (no foreign-born individuals in the study populations from Russia and Slovakia; intermediate percentages from Madrid, Spain (30.8%), and Greece (39.7%), and higher rates in Genova, Italy (69.6%), Barcelona, Spain (72.7%), Rome, Italy (74.6%), France (78.4%), and the United Kingdom. HIV prevalence was available from 8 centers and ranged from 0% (Greece) to 15.2% (Russia).

**Table 1.** Prevalence of Diabetes Mellitus Among Patients With Tuberculosis Diagnosed in 13 European Clinical Centers and Contribution of Centers to a Case-Control Study

Center	Years^b^	Prevalence Study								Case-Control Study	
		TB, No.	Male Gender, %	Age, Median, y	Foreign- Born, %	HIV+, %	DM, No.	TB-DM/TB%, 95% CI	DM General Population Prevalence^a^	Cases, No.	Controls, No.
France	2010–2012	116	83.6	45	78.4	nr	9	7.8, 3.6–14.2	5.2	9	27
Germany	2012–2015	163	65.0	44	66.9	nr	15	9.2, 5.2–14.7	7.9	15	45
Greece	2010–2014	68	73.5	43	39.7	0.0	3	4.4, 0.9–12.3	4.8	3	9
Italy–Genova	2009–2011	79	74.7	38	69.6	8.9	8	10.1, 4.5–19.0	4.9	8	24
Italy–Rome	2007–2012	956	65.3	36	74.6	14.7	63	6.6, 5.1–8.4	4.9	63	189
Norway	2009–2013	Data not available								12	36
Russia	2009–2013	374	78.1	40	0.0	15.2	45	12.0, 8.9–15.8	5.0	45	135
Slovakia	2015	101	79.2	49	0.0	nr	10	9.9, 4.9–17.5	7.2	10	30
Spain–Barcelona	2009–2013	198	81.8	37	72.7	11.6	24	12.1, 7.9–17.5	7.9	24	72
Spain–Pontevedra	2009–2013	394	61.7	46	5.6	4.1	26	6.6, 4.4–9.5	7.9	12	36
Spain–Madrid	2009–2013	266	65.0	42	30.8	12.0	12	4.5, 2.4–7.8	7.9	26	78
Ukraine	2009–2013	Data not available								27	81
United Kingdom	2012–2015	428	64.7	36	85.3	2.1	122	28.5, 24.3–33.0	3.9	71	213

Abbreviations: CI, confidence interval; DM, diabetes mellitus; TB, tuberculosis.

^a^International Diabetes Atlas – 2014.

^b^Diagnosis periods in case-control studies differ slightly with respect to prevalence period for France and Greece, 2011–2012 and 2010–2013, respectively.

**Figure 1.**
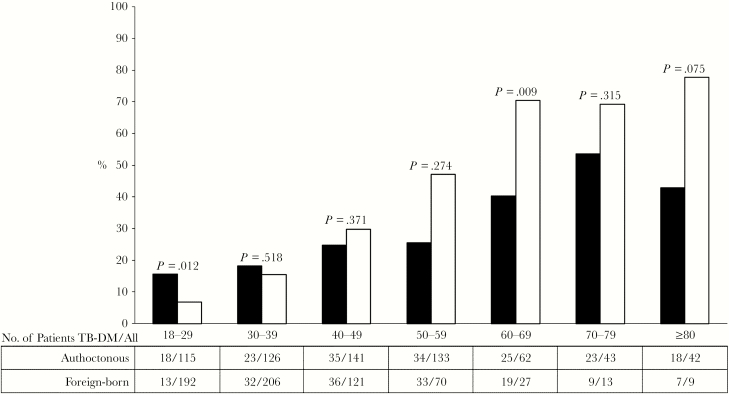
Prevalence of diabetes mellitus (DM) among patients with tuberculosis (TB) by age and migration status.

Of the patients included in the analysis, 337 were diagnosed with DM, and the overall prevalence was 10.7% (95% CI, 9.7%–11.9%); in different countries, DM prevalence ranged from 4.4% in Greece to 28.5% in the United Kingdom. No significant difference was found between the 2 centers in Italy, whereas the prevalence varied significantly among the 3 Spanish centers (*P* < .05 by chi-square test). No significant trends over time in DM prevalence in participating centers were observed (data not shown). DM prevalence increased with age, more markedly among foreign born (Figure 1).

Among 325 TB-DM cases for whom information was available, 21.8% were newly diagnosed DM cases. DM was more frequently diagnosed at the time of TB diagnosis in foreign-born than autochthonous patients (43/148, 29.0%, vs 28/177, 15.8%; *P* = .004). Information on DM treatment was available for 271/325 (83.4%) DM cases, and 91.9% were in treatment. One-half of patients were treated with oral hypoglycemic drugs and the others with insulin.

### Associations Between Diabetes in Tuberculosis and Clinical and Demographic Characteristics

To analyze the association between DM and demographic and clinical characteristics, we compared 325 TB-DM cases and 975 TB-only controls diagnosed consecutively after each TB-DM case in the same center (total 1300) (Table 1). Pulmonary and extrapulmonary TB disease were equally distributed among cases and controls (pulmonary localization: 229/325, 70.5%, vs 687/975, 70.5%; extrapulmonary localization: 72/325, 22.2%, vs 201/975, 20.6%; both pulmonary and extrapulmonary localizations: 24/325, 7.4%, vs 87/975, 8.9%).

In univariable analysis (Table 2), DM cases were significantly older (odds ratio [OR] for age ≥50 years, 3.6; 95% CI, 2.1–6.2; *P* < .001) and more likely to have comorbidities (OR, 1.6; 95% CI, 1.0–2.6; *P* = .058), whereas they were less likely to originate from foreign countries (OR, 0.7; 95% CI, 0.5–1.1; *P* = .153). There was no evidence of an association between DM and HIV infection, country of birth prevalence, previous TB disease, excessive alcohol intake, and drug abuse.

**Table 2.** Case-Control Study on Tuberculosis in Diabetes Mellitus in Patients Enrolled in 13 European Centers

	TB-DM (n = 325), No. (%)	TB (n = 975), No. (%)	Univariable^a^			Multivariable^a^	
			OR (95% CI)	*P*	Interaction Term	OR (95% CI)	*P*
Age class, y							
<50	157 (48.3)	744 (76.3)	1		Authoctonous	1	
					Foreign-born	0.9 (0.5–1.7)	.687
≥50	168 (51.7)	231 (23.7)	3.6 (2.1–6.2)	<.001	Authoctonous	1	
					Foreign-born	2.8 (1.7–4.7)	<.001
Migration status							
Authoctonous	176 (54.1)	486 (49.9)	1		Age < 50 y	1	
					Age ≥ 50 y	2.3 (1.1–4.8)	.026
Foreign-born	149 (45.9)	489 (50.1)	0.7 (0.5–1.1)	.153	Age < 50 y	1	
					Age ≥ 50 y	7.3 (5.1–10.5)	<.001
Country of birth by DM prevalence^b^							
≤6.5%	107 (32.9)	321 (32.9)	1			1	
6.6%–8.3%	119 (36.6)	313 (32.1)	1.3 (0.8–2.0)	.313		1.3 (0.7–2.3)	.424
≥8.4%	99 (30.5)	341 (35.0)	0.8 (0.5–1.3)	.357		0.8 (0.5–1.2)	.231
Gender							
Female	90 (27.7)	290 (29.7)	1			1	
Male	235 (72.3)	685 (70.3)	1.1 (0.8–1.6)	.607		1.0 (0.6–1.6)	.920
Previous TB disease							
No	247 (76.0)	767 (78.7)	1				
Yes	53 (16.3)	130 (13.3)	1.3 (0.8–2.1)	.353			
Not available	25 (7.7)	78 (8.0)	1.0 (0.7–1.5)	.987			
Heavy drinking							
No	222 (68.3)	636 (65.2)	1				
Yes	38 (11.7)	122 (12.5)	0.9 (0.6–1.3)	.536			
Not available	65 (20.0)	217 (22.3)	0.7 (0.4–1.3)	.259			
Injecting drug users							
No	232 (71.4)	725 (74.4)	1				
Yes	14 (4.3)	44 (4.5)	1.0 (0.3–3.7)	.976			
Not available	79 (24.3)	206 (21.1)	2.2 (0.9–5.3)	.093			
HIV status							
Negative	255 (78.5)	779 (79.9)	1				
Positive	23 (7.1)	84 (8.6)	0.8 (0.2–3.1)	.776			
Not available	47 (14.5)	112 (11.5)	1.4 (1.0–1.8)	.025			
Other comorbidities							
No	231 (71.1)	697 (71.5)	1			1	
Yes	70 (21.5)	137 (14.0)	1.6 (1.0–2.6)	.058		1.1 (0.7–1.6)	.711
Not available	24 (7.4)	141 (14.5)	0.4 (0.1–1.8)	.235		0.3 (0.1–1.5)	.144

Abbreviations: CI, confidence interval; DM, diabetes mellitus; OR, odds ratio; TB, tuberculosis.

^a^Conditional logistic regression model with robust standard error estimated by including the center as the cluster variable.

^b^International Diabetes Atlas – 2014.

The multivariable conditional regression model with an interaction term between age and migration status had a better fit than the model without it. Other interactions were not significant. The final multivariable model, in addition to age, migration status, and their interaction, included comorbidities also and was adjusted for country of birth by DM prevalence and gender. Multivariable analysis confirmed the association between DM and older age both among authoctonous and among foreign-born (OR, 2.3; 95% CI, 1.1–4.8; *P* = .026; OR, 7.3; 95% CI, 5.1–10.5; *P* < .001, respectively). Among older patients DM was associated with being foreign-born rather than authoctonous (OR, 2.8; 95% CI, 1.7–4.7; *P* < .001). An association with comorbidities was not confirmed (Table 2).

To analyze the association between DM and radiological and clinical characteristics of pulmonary TB, we compared 254 cases and 762 controls. At univariable analysis, a positive sputum culture was significantly associated with DM (OR, 1.4; 95% CI, 1.1–1.7; *P* = .003); moreover, DM diagnosis was more likely among patients with radiological evidence of cavities (OR, 1.6; 95% CI, 1.0–2.5; *P* = .054), with a positive sputum smear microscopy (OR, 1.4; 95% CI, 1.0–2.2; *P* = .077), and among those reporting the presence of persisting cough (OR, 1.4; 95% CI, 0.9–2.0; *P* = .086). In the multivariable analysis, the presence of persisting cough and radiological evidence of cavities were more frequent among patients with DM, altough no association that was significant at the *P* < .05 level was observed. (Table 3).

**Table 3.** Case-Control Study on Tuberculosis in Diabetes Mellitus in Patients Enrolled in 13 European Centers—Analysis of Radiological and Clinical Characteristics Among Patients With Pulmonary Involvment

			Univariable^a^		Multivariable^a b^	
	TB-DM (n = 254), No. (%)	TB (n = 762), No. (%)	OR (95% CI)	*P*	OR (95% CI)	*P*
TB site of disease						
Pulmonary	228 (89.8)	681 (89.4)	1			
Pulmonary and extrapulmonary	26 (10.2)	81 (10.6)	0.9 (0.6–1.5)	.857		
CXR cavities						
No	94 (37.0)	341 (44.7)	1		1	
Yes	146 (57.5)	365 (47.9)	1.6 (1.0–2.5)	.054	1.6 (0.9–2.8)	.110
Not available	14 (5.5)	56 (7.4)	0.8 (0.3–1.9)	.584	1.0 (0.5–2.1)	.992
Sputum smear microscopy–positive						
No	77 (30.3)	283 (37.1)	1		1	
Yes	156 (61.4)	416 (54.6)	1.4 (1.0–2.2)	.077	1.3 (0.7–2.7)	.425
Not available	21 (8.3)	63 (8.3)	1.2 (0.6–2.4)	.558	1.2 (0.5–2.9)	.667
Sputum culture–positive						
No	26 (10.4)	100 (13.1)	1		1	
Yes	201 (79.1)	577 (75.7)	1.4 (1.1–1.7)	.003	0.9 (0.5–1.8)	.888
Not available	27 (10.6)	85 (11.1)	1.1 (0.8–1.6)	.416	1.1 (0.6–2.0)	.676
Weight loss						
No	109 (42.9)	315 (41.3)	1			
Yes	127 (50.0)	342 (48.9)	1.1 (0.6–1.8)	.794		
Not available	18 (7.1)	105 (13.8)	0.4 (0.1–1.3)	.117		
Persisting cough						
No	42 (16.5)	146 (19.2)	1		1	
Yes	199 (78.3)	523 (68.6)	1.4 (0.9–2.0)	.086	1.5 (0.9–2.3)	.092
Not available	13 (5.1)	93 (12.2)	0.4 (0.1–2.0)	.261	0.5 (0.1–1.7)	.248

Abbreviations: CI, confidence interval; CXR, chest x-ray; DM, diabetes mellitus; OR, odds ratio; TB, tuberculosis.

^a^Conditional logistic regression model with robust standard error estimated by including the center as the cluster variable.

^b^Adjusted for age, gender, and HIV status.

## DISCUSSION

This multicenter study collected DM prevalence data from a large population of TB patients attending 11 specialized clinical centers in Europe and found a general overall prevalence higher than the reported comparative DM prevalence in the general European population [10]. This result should nevertheless be considered cautiously, as the studied population was not a random sample of patients diagnosed with TB in countries where the study was carried out.

A wide variation of DM prevalence was reported across countries (4.4% in Greece to 28.5% in the United Kingdom) and across some centers (4.5% to 12.1% in 3 centers in Spain) with different annual caseloads; nevertheless, most centers, as expected, reported a DM prevalence higher than the comparative DM prevalence for the general population of each respective country and higher than previous findings from single-center or nationwide population-based studies on TB-DM patients performed in Europe [15, 20–28]. One study conducted in Madrid [20] reported a DM prevalence almost double (8.6%) that collected by our study site in Madrid (4.5%). The population analyzed by Fortún et al. [20] was nevertheless larger and recruited during a more extended period. The highest DM prevalence rate was reported in the United Kingdom, where it was 4 times greater than comparative DM prevalence for the UK general population. This could be explained by the specific characteristics of this study population, including the extremely high proportion of foreign-born patients. Differences in diagnostic practices for DM may also contribute to differences in DM prevalence in different centers. Taken together, however, these studies suggest that DM patients constitute a relevant proportion of individuals with TB in European countries.

Older age was identified as a risk factor for TB-DM comorbidity, in agreement with a recent systematic review [11] and with other European studies [15, 23, 25]. This association may reflect the current distribution of DM among the general population in Europe. Moreover, older-aged (≥50 years) foreign-born DM prevalence was approximately 3 times higher than that among authoctonous individuals in that age group.

An extensive systematic review has identified both male [29] and female [11] gender as risk factors for TB-DM comorbidity. We could not find any association with gender.

The proportion of foreign-born TB patients varied widely across TB reference centers, and this could reflect differences in patterns of migration and population structure among the participating countries (ie, the United Kingdom, France; Italy vs Russia or Slovakia) or when differences were found in the same country (in the case of Spain) or among different cities in the same country. Foreign-born TB patients were more likely to have DM compared with authoctonous individuals if aged ≥50 years, independent of DM prevalence in their country of origin, consistent with previous studies from high-income countries [25, 30, 31]. People migrating from low- and middle-income countries may have a higher prevalence of DM compared with the native population, and also somehow higher than in persons from the same population who remain in their country of origin [32]. Nevertheless, opposite results (ie, lower risk of DM in foreign-born TB patients compared with authoctonous individuals or lower risk of TB in foreign-born DM patients compared with authoctonous) have been reported by previous studies conducted in Italy [24], Spain [15, 17], Portugal [23], and Japan [33].

As in other studies, DM was mainly diagnosed before a TB diagnosis in our patient population. However, approximately one-fifth of the patients with DM, most of whom were foreign-born, were identified at the time of TB diagnosis, possibly reflecting difficulties in accessing or obtaining specialized medical care. Poor glycemic control has been shown to increase the risk of TB for patients with DM [34], so we could hypothesize that the high proportion of undiagnosed DM, especially among foreign-born patients, may be itself a further factor contributing to the risk of developing TB in this population. The existence of a sizeable proportion of TB patients in whom DM was not diagnosed until TB diagnosis, who were possibly at risk of uncontrolled DM, emphasizes the relevance of DM screening, especially in patients migrating from high–TB burden countries. A policy of bidirectional low-cost screening (screening DM patients for TB and TB patients for DM) would be an effective way of identifying a vulnerable population for both diseases promptly.

The relatively higher level of incidence and prevalence of diabetes among people living with HIV, and particularly among those who are receiving antiretroviral treatment, warrants the screening of people living with HIV for hyperglycemia, both at time of enrollment and during the follow-up period of HIV treatment [35]. Nevertheless, the complex interaction among DM, HIV, and TB is still unclear: A case-control study from Tanzania identified DM as a risk factor for TB in the non-HIV-infected, but not in HIV-infected patients [36]. In our study, no association with HIV infection was identified. A negative association between being HIV-positive and DM prevalence among TB patients was reported by other studies also conducted in Europe—Italy [25], Spain [15, 17], and Portugal [23]. It has been suggested that the very strong association between HIV and TB may obscure the role of DM [6, 37]; however, a recent study concluded that DM may further increase the risk of developing TB among persons living with HIV [38].

DM has been found to be an independent risk factor for higher prevalence or greater severity of some symptoms such as cough, hemoptysis, fever, and delayed sputum conversion [39–41]. Data indicating higher bacillary burden in sputa are conflicting [42–44]. In our study, no significant differences in the prevalence of symptoms were recorded among the totality of TB patients with pulmonary involvement, with or without DM. Previous studies show that cavities on chest x-rays are significantly more frequent among TB-DM patients [15, 25, 45], suggesting that DM patients with TB are on average more infectious than those without DM [46]. In our study, pulmonary cavities were more frequent among DM patients than those without DM, although this difference was not statistically significant.

### Limitations

Due to its voluntary nature and retrospective and multicentric design, our study has some methodological limitations. Representativeness and reliability of DM prevalence data are limited due to the participation of few countries and to the relatively small sample. Missing data on HIV status and the heterogeneity of recruited populations among centers with foreign-born patient proportions may have caused over- or underestimation of some associations. Partial duplication of data has to be acknowledged for 1 of the Italian study centers (Rome) [45]. Despite planning at the protocol stage, the information obtained on glucose test results and glycemic control was incomplete. TB outcomes, income status, and urban residency were not investigated.

## CONCLUSIONS

To our knowledge, this is the first multicenter study reporting on DM in TB patients in Europe, where DM represents a challenge for TB control. Older patients and foreign-born individuals emerged as vulnerable populations, and this suggests that they should be a priority target for TB screening among those with DM.

Further studies to analyze the association of DM with drug resistance and with TB outcomes, to evaluate whether TB-DM patients contribute to enhanced TB transmission in low–TB burden countries, and to obtain broader cross-sectional and prospective data that may help in assessing the feasibility and cost-effectiveness of TB-DM bi-directional screening are needed.

## Notes

Prior presentation: Results of this study were presented in part at the European Respiratory Society International Congress (September 3–7, 2016; London, UK) as a poster presentation: “Socio-demographic and Clinical Characteristics of Patients With Tuberculosis and Diabetes in European countries. A TBnet Study.”
